# Design, Fabrication and Experimental Validation of a Novel Dry-Contact Sensor for Measuring Electroencephalography Signals without Skin Preparation

**DOI:** 10.3390/s110605819

**Published:** 2011-05-30

**Authors:** Lun-De Liao, I-Jan Wang, Sheng-Fu Chen, Jyh-Yeong Chang, Chin-Teng Lin

**Affiliations:** 1 Department of Electrical Engineering, National Chiao Tung University, Hsinchu 300, Taiwan; E-Mails: gs336.tw@gmail.com (L.-D.L.); jychang@mail.nctu.edu.tw (J.-Y.C.); 2 Brain Research Center, University System of Taiwan, Hsinchu 300, Taiwan; E-Mail: double0105@gmail.com (I-J.W.); 3 Department of Computer Science, National Chiao Tung University, Hsinchu 300, Taiwan; 4 Division of Medical Engineering Research, National Health Research Institutes, Miaoli 350, Taiwan; E-Mail: sanfo@nhri.org.tw (S.-F.C.); 5 Institute of Imaging and Biomedical Photonics, National Chiao Tung University, Hsinchu, Taiwan

**Keywords:** electroencephalography (EEG), dry electrode, conductive gels, skin-sensor interface impedance

## Abstract

In the present study, novel dry-contact sensors for measuring electro-encephalography (EEG) signals without any skin preparation are designed, fabricated by an injection molding manufacturing process and experimentally validated. Conventional wet electrodes are commonly used to measure EEG signals; they provide excellent EEG signals subject to proper skin preparation and conductive gel application. However, a series of skin preparation procedures for applying the wet electrodes is always required and usually creates trouble for users. To overcome these drawbacks, novel dry-contact EEG sensors were proposed for potential operation in the presence or absence of hair and without any skin preparation or conductive gel usage. The dry EEG sensors were designed to contact the scalp surface with 17 spring contact probes. Each probe was designed to include a probe head, plunger, spring, and barrel. The 17 probes were inserted into a flexible substrate using a one-time forming process via an established injection molding procedure. With these 17 spring contact probes, the flexible substrate allows for high geometric conformity between the sensor and the irregular scalp surface to maintain low skin-sensor interface impedance. Additionally, the flexible substrate also initiates a sensor buffer effect, eliminating pain when force is applied. The proposed dry EEG sensor was reliable in measuring EEG signals without any skin preparation or conductive gel usage, as compared with the conventional wet electrodes.

## 1. Introduction

Measuring electrocardiogram (ECG), electroencephalogram (EEG), electrooculogram (EOG), and electromyogram signals is significant for both research and clinical applications, especially in the case of EEG data [1]. EEG is a method of measuring the electrical activity of the brain using electrodes placed along the scalp skin [1,2]. Because EEG is a powerful and noninvasive tool that can provide high temporal resolution to directly reflect the dynamics of brain activities [3,4], it has been widely used for both medical diagnoses and neurobiological research [1,5–8]. Conventional wet Ag/AgCl electrodes are generally and most frequently used to measure EEG signals [1]. The conventional wet electrode characteristics have been widely studied and discussed in detail, including their applications [9]. Indeed, EEG signal quality is excellent with the proper skin preparation and conductive gel usage.

However, skin preparation and the use of conductive gels are always required when using conventional wet electrodes. These processes are employed to reduce skin-sensor interface impedance [10]. In terms of the convenience of the EEG signal measurement process, these procedures usually create trouble for users [11], especially in daily life applications for long-term nonitoring. In particular, the use of conductive gels inevitably leaves residues on the scalp. The gel may also leak out of the wet EEG electrodes, causing a short circuit between two electrodes in close proximity when too much gel is applied or the wet electrode is pushed down too hard on the scalp [12]. Moreover, the above-mentioned preparation procedures for wet electrodes also have some significant drawbacks, such as being time-consuming, uncomfortable, and painful for participants because the skin preparation usually involves outer skin layer abrasion.

Repeated skin preparations and gel applications may also induce allergic reactions or infections. The EEG signal quality may degrade over extensive time periods as the skin regenerates and/or the conductive gel dries [11]. Some issues also arise when measuring a location of interest that is covered with hair. This procedure leads to insufficient skin-electrode contact area, especially for long-term studies.

To improve the performance of conventional wet EEG sensors, micro-electro-mechanical systems (MEMS) have been used to develop dry MEMS electrodes to measure EEG signals [13–16]. Dry MEMS EEG electrodes with several micro-needles on their top surface can successfully acquire forehead EEG signals [13,14,16]. The micro-needles are used to penetrate the outer skin surface layers to acquire the EEG signals [16]. The characteristics of this kind of dry MEMS EEG electrode have also been discussed in detail [17], including comparisons to the equivalent circuits of the wet electrode-skin interface. Dry MEMS electrodes can potentially be used to acquire EEG signals without skin preparation or the use of conductive gels. However, there is no convincing evidence about EEG signal quality under hairy or hairless sites using dry MEMS EEG electrodes. Moreover, some disadvantages to using dry MEMS electrodes remain, such as the lack of physical strength with which the micro-needles attach to the skin surface. Recently, Ruffini *et al.* demonstrated carbon nanotube (CNT)-based dry electrodes for applications in biopotential signal studies [14,18]. These authors also measured biopotential signals in human trials for the first time [18]. Recently, Grozea *et al.* proposed another type of dry MEMS sensor, the bristle-sensor, for EEG measurement [19]. They indicate that the performance of the bristle-sensors compares well with wet electrodes [19]. The success of this prototype means that it is possible to measure EEG signals on hairy sites by dry MEMS-linked electrodes.

Recently, fabric-based electrodes have been used to measure biopotential signals [20–24]. Beckmann *et al.* tailored their fabric electrodes with different fabric specifications to record ECG signals in detail [23]. Our recent study successfully utilized foam-based electrodes that were covered by conductive fabric materials to acquire forehead EEG signals for the first time [25]. Compared with MEMS-based electrodes for measuring biopotential signals, the fabric-based electrodes are relatively comfortable and noninvasive. However, the fabric-based electrodes used to record EEG signals on hairy sites require further improvement because the hairs reduce the contact area at the skin-electrode interface.

In contrast to previous dry electrode types, Matthews *et al.* proposed a hybrid dry electrode for recording EEG measurements [26–28]. These electrodes combine high-impedance resistive, capacitive characteristics and contact the scalp without any skin preparation, and depend on a high contact impedance between the scalp and the electrode. However, these electrodes possess hard substrates, which lead to discomfort or even pain on the scalp surface when force is applied. The EEG signal may be distorted easily by motion due to the hard substrate [9]. Moreover, the fabrication cost for a high-contact impedance electrode is higher than for the other types of dry EEG electrodes.

In addition to the dry-contact electrode types, noncontact (capacitive) electrodes have also demonstrated the potential to acquire EEG signals with spacing between the electrode and body and no skin preparation [29–31]. Matthews *et al.* proposed a dry capacitive electrode to noninvasively measure EEG signals without skin preparation [31]. However, dry capacitive electrodes are easily affected by motion artifacts [9].

In this study, a novel dry-contact sensor is demonstrated for measuring EEG signals without skin preparation or conductive gel usage, even on hairy sites. This dry EEG sensor consists of a spring contact probe, a flexible substrate, and a one-time sensor-forming process via the injection molding procedure. The results demonstrate that our proposed dry EEG sensor could be used to measure EEG signals without skin preparation or conductive gel usage. Moreover, the dry EEG sensor flexible substrate with several spring probes allows for high geometric conformity between the electrode and the irregular scalp surface to maintain low skin-electrode interface impedance. This technique complements other existing EEG measurement approaches for the investigation of human EEG states involving neuronal activation and the behavioral responses to daily life applications [32,33].

## 2. Materials and Methods

### 2.1. Novel Dry-Contact Sensor Design

**Figure 1.**
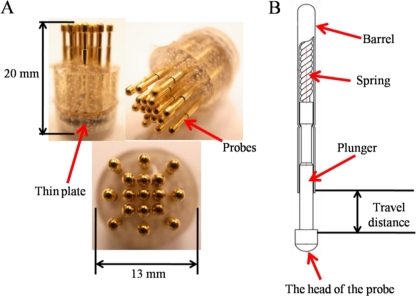
(**A**) Several images of the proposed dry EEG sensor are shown. (**B**) An exploded view of the proposed dry sensor is presented. Each probe includes a probe, plunger, spring, and barrel.

The proposed dry EEG sensors were designed and fabricated (in cooperation with CCP Contact, Ltd., Taiwan) to contact the scalp surface with 17 “probes,” as shown in Figure 1(A). Each probe was designed to include four components: (1) probe head, (2) plunger, (3) spring, and (4) barrel (Figure 1(B)).

The probe head was a fabricated spheroid with a radius of 1.3 mm, which was coated with gold (Au) for enhanced conductivity. Moreover, Au is biocompatible and does not produce adverse effects or reactions on the skin-sensor interface. To acquire effective EEG signals on hairy sites, the probe head size should be properly designed to pass through the hairs without trapping hair beneath the probe and thereby preventing electrical contact with the scalp. The probe heads were custom-made to be larger than the pores of the scalp (<0.5 mm) so that the probes did not penetrate into the skin and cause pain when the probes were tightly attached to the scalp surface. Next, the probe heads were designed to be embedded into the plungers. The plungers were made of beryllium copper (BeCu) and were optimized during the turning and plating processes, resulting in straight and exact plunger surfaces, which will enable a long lifetime. Each plunger was connected to a spring with 20 g of feedback force buffer. The spring was covered by the outside barrel, as shown in Figure 1(B). This mechanism was designed to allow a feedback force of 20 g to act as a “buffer” for the probe when a force was applied to the dry sensors. The spring force selection of approximately 20 g depended mainly on the scalp EEG measurement application. The spring force needed to be large enough to ensure that the sensors could attach to the scalp surface and maintain the proper sensor-skin contact impedance quality. The spring force should also not lead to any damages on the contacting surface. The compression springs were made of silver-plated music wire, and for measuring special EEG signals, nonmagnetic BeCu was employed. Springs composed of music wire had a working temperature of up to 80 °C (176 °F), while stainless-steel and BeCu springs could be operated up to 250 °C (482 °F) and 200 °C (392 °F), respectively. The increases in the spring force were designed to be proportional to the spring travel distance (4 mm). During probe assembly, the spring was already compressed by a certain travel distance. The resulting spring force is called preload. The preload effects ensure that there is a certain force from the beginning of the contacting process and also that the plunger is completely pushed back after contacting the scalp surface. After assembly of the spring contact probes, the integrated probes can be used more than 100,000 times.

**Figure 2.**
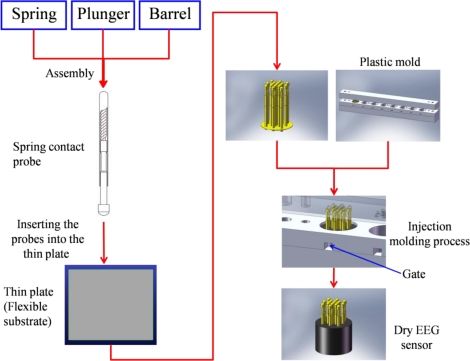
The fabrication process of novel dry EEG sensors.

Seventeen probes were next inserted into the thin Cu plate that served as the flexible substrate of the sensor. The circular thin Cu plate was constructed with a thickness of 2 mm and a diameter of 13 mm (*i.e.*, flexible substrate). After insertion, all of the probes on the thin plate were conductive together. In addition, the plate thinness leads to a flexible substrate characteristic when force is applied. When a force is applied on the sensor, the flexible substrate fits the scalp interface well, as shown in Figure 1(A). The spring contact probes and flexible substrate both provide buffering effects, enabling the dry EEG sensor to attach well to the scalp with no pain when force is applied. After probe fabrication and insertion into the flexible substrate, an established injection molding process was used to form the flexible substrate with several probes, which was necessary only one time, as shown in Figure 2. The probes with the flexible substrate were fixed in the mold, and then the soft enclosing silicone materials were injected into the mold to encase the sensor.

**Figure 3.**
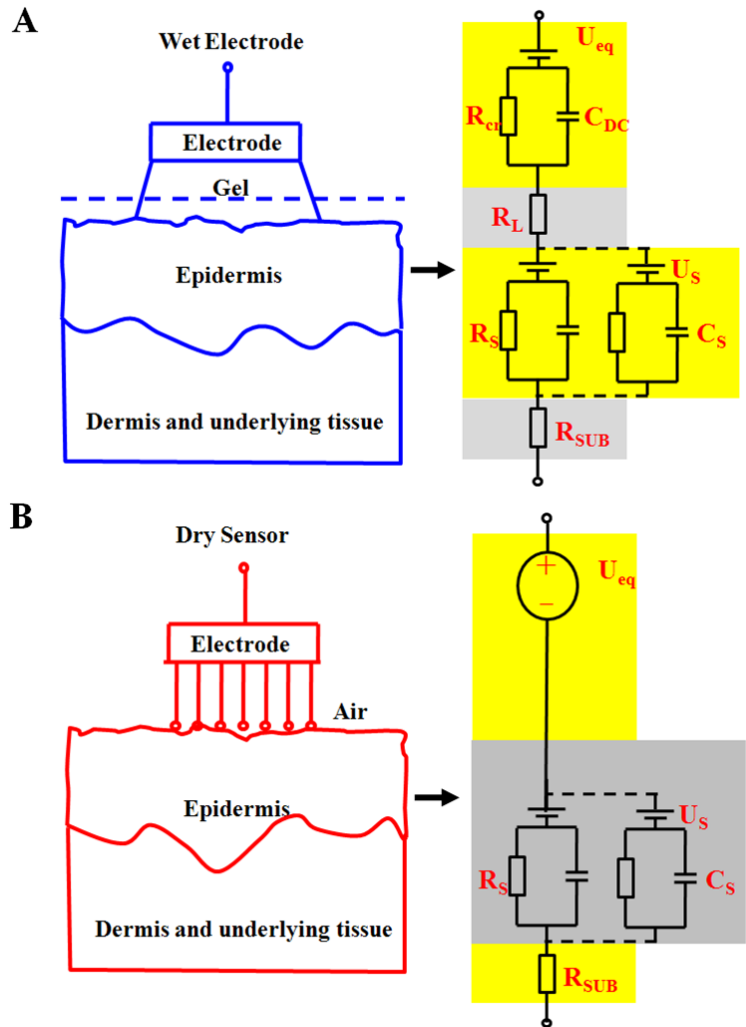
The equivalent circuits of (**A**) a wet electrode-skin interface and (**B**) a dry skin-sensor interface.

Traditionally, oxidation and reduction reactions are strongly affected by wet electrodes and occur at the metal-electrolyte interface. In Figure 3(A), the equivalent circuit of the wet electrode consists of a half-cell potential (*U*_eq_), a double-layer capacitance (*C*_DC_), and parallel and series resistances (*R*_Cr_). Human skin includes epidermis, dermis, and subcutis layers. These layers can be simulated by a capacitor, *C_s_*. The numerous channels, preparatory glands, and hair follicles within the skin connect the layers and are represented by the resistance *R_s_*. The subcutis layer is well supplied with blood and may therefore be represented as the resistance *R*_SUB_. The layered structure of the stratum corneum leads to semipermeable membranes and ion concentration differences, which results in a potential, *U_s_*.

As with conventional wet electrodes, the proposed dry EEG sensor exhibits conductivity only because of its electrically conductive probes [34]. The high conductivity behavior at the sensor-skin interface can be measured by the proposed dry sensors [35]. The equivalent circuit model of the proposed dry EEG sensor skin-electrode interface is shown in Figure 3(B) [9,34]. Biopotentials can be measured directly with the conductive method, and the influence of the stratum corneum can be effectively reduced [9]. Moreover, sweat and skin humidity can also form a conductive path, *R_L_* [36]. The design of the dry EEG sensors can fit the scalp surface well to increase the contact area between the skin and sensor to reduce the impedance, *R_L_*. If the impedance of the EEG sensor is kept in a proper range between the sensor and skin interface, the EEG signals can be detected due to a proper contact impedance [2,35].

### 2.2. Wireless EEG Acquisition Readout Circuit

**Figure 4.**
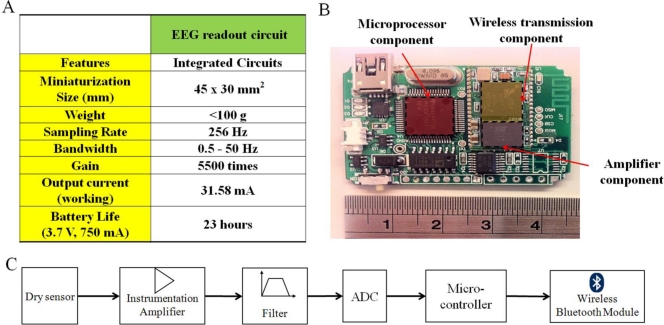
(**A**) The wireless EEG acquisition module and the performance characteristics with (**B**) a top view of the readout circuit and (**C**) the design diagram in differential configuration are shown.

Figure 4 presents the wireless EEG acquisition module. The module was used to acquire EEG signals from the proposed dry EEG sensors and includes a biosignal amplifier (INA2126, Texas Instruments, USA), an acquisition component (AD8609, Analog Devices, USA), a microprocessor component (MSP430, Texas Instruments), and a wireless transmission component (BM0403, Unigrand Ltd., Taiwan) (Figure 4(B)) [32]. To amplify and filter the EEG signals, a preamplifier, a band-pass filter (0.5–50 Hz), and an analog-to-digital converter (ADC) were embedded into our circuit board as biosignal amplifier and acquisition component modules (Figure 4(C)).

The amplifier and acquisition component gains were set to approximately 5,500, with a frequency band from 0.5 to 50 Hz. An ADC with 12-bit resolution was used to digitize the EEG signals, and a sampling rate of 256 Hz for the amplified and filtered EEG signals was employed. In the microprocessor component, the EEG signals that were probed using an ADC were digitally stored. A moving average filter with a frequency of 60 Hz was then applied to reject any power-line interference before the wireless transmission. A Bluetooth module, BM0403 (Unigrand Ltd.), was included in the wireless transmission portion of the circuit. Notably, the module was fully compliant with the specifications for a Bluetooth v2.0+ enhanced data rate (EDR) and a printed circuit board (PCB) antenna. Finally, the designed wireless EEG acquisition module size was approximately 4.5 × 3 × 0.6 cm^3^. This module was operated continuously for 23 h at 31.58 mA (which includes the 20 mA from the wireless part, 330 μA from the microprocessor and 0.71 mA from the amplifier component) with a 3.7-V DC power supply and a commercial 750 mAh Li-ion battery. This wireless EEG acquisition module was used to measure the EEG signals with wet electrodes and dry EEG sensors in the following experiments.

## 3. Results and Discussion

### 3.1. Dry EEG Sensor Performance

**Figure 5.**
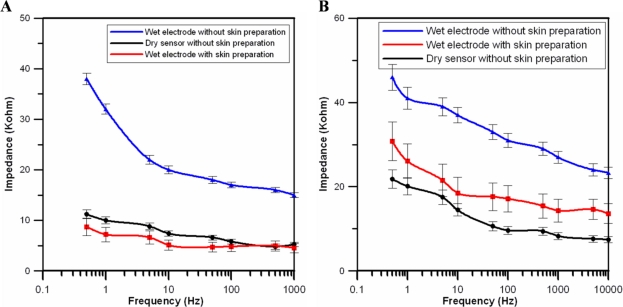
Impedance change data representing the skin-electrode interface on the (**A**) forehead (F10) and (**B**) at hairy sites (POz).

The impedance of the skin-sensor interface was analyzed by impedance spectroscopy (LCR4235, Wayne Kerr Electronics Ltd., UK). Two dry electrodes were placed on the forehead (F10) (less than 1 cm apart), and then a voltage was applied to the electrode pair to measure the impedance changes [35,37]. Before the experiments, the skin of the subject was cleaned carefully according to standard skin preparation procedures on the sites for the wet electrodes only. To guarantee reliable and reproducible results, the test signal for the impedance spectroscopy was set to 1 V and the frequency range was from 0.5 to 1,000 Hz. Twenty tests were performed on five different subjects in this study. Figure 5(A,B) show the averaged values and standard deviations of the impedance results under different conditions.

The black solid line denotes the impedance changes of our dry EEG sensor pair without skin preparation and without the use of conductive gel, as shown in Figure 5(A). The blue and red solid lines denote the impedances of the conventional wet electrodes with and without skin preparation, respectively. In addition, all of the conventional wet electrodes were applied with conductive gels. The results show that the measured impedance changes between the skin and the dry EEG sensors were similar to those of the conventional wet electrodes with conductive gels. Figure 5(B) reports the impedance measurements on a hairy site (POz). The results indicate that the impedance of the proposed dry EEG sensor is close to that of the wet electrode and is even lower on the hairy site. Significantly, the flexibility of the proposed dry EEG sensor is effective in tightly contacting the scalp surface and providing clear EEG signals without any skin preparation or conductive gel usage.

**Figure 6.**
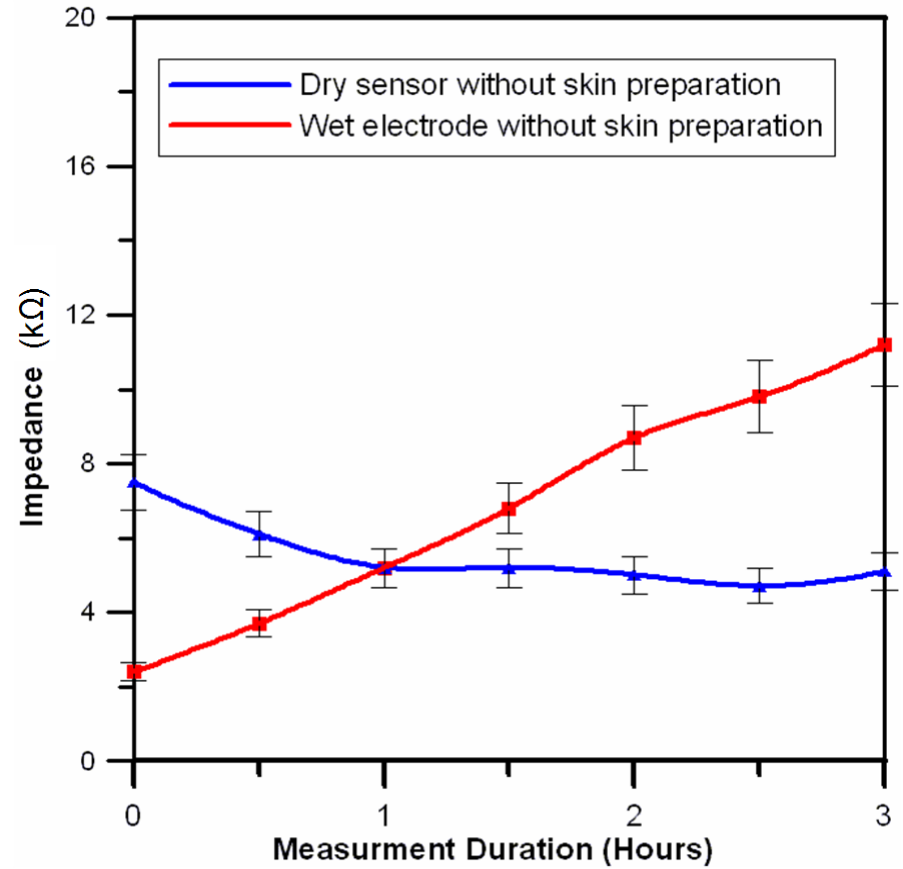
Long-term impedance variation measurements on the forehead site (F10) for wet and dry electrodes.

Figure 6 shows the averaged long-term impedance variation values (3 h) for six subjects. The long-term impedance variations of the conventional wet electrodes with conductive gels were significantly larger than those of the dry EEG sensor. The impedance variation of the dry EEG sensor was in the range from 4 to 14 kΩ, which is in the acceptable range for normal EEG measurements [11,37,38]. Furthermore, the dry sensors can provide significantly enhanced stability of the skin-sensor interface impedance because conductive gels, which are apt to drying, are not required [22].

### 3.2. EEG Signal Quality and Comparison Using the Wet Electrodes and Proposed Dry Sensors

**Figure 7.**
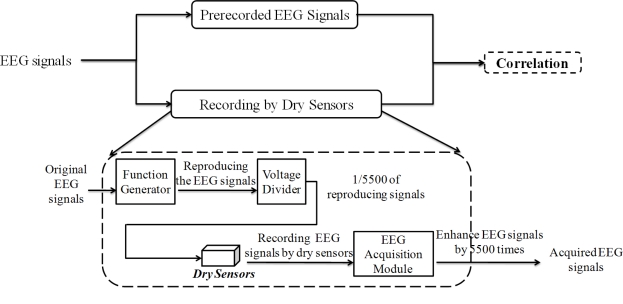
Experimental process for signal quality assurance of the dry sensors.

**Figure 8.**
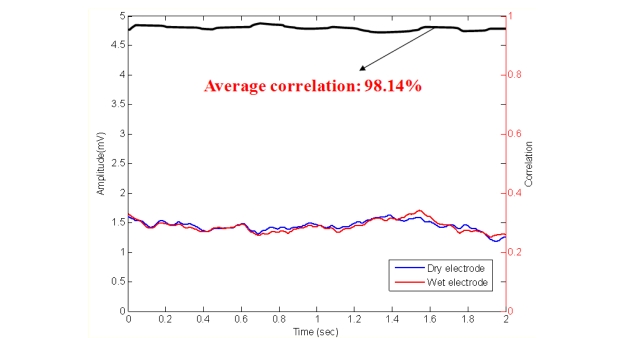
Prerecorded EEG and corresponding signals recorded by the dry EEG sensor.

To assure the EEG signal quality, an experiment was performed prior to those described above to determine the distortion caused by our dry EEG sensors, as shown in Figure 7. In the beginning of the process, EEG data were prerecorded with 512 Hz sampling rates using standard EEG electrodes that were equipped with conductive gels. The data were digitally stored in a computer. Next, the EEG data were fed to a programmable function generator and passed through a voltage divider to generate the simulated EEG signal from a real human brain. The simulated EEG signal was further fed to the dry EEG sensors and then amplified by the EEG machine. After recording the amplified EEG signal, the signal was compared with the prerecorded EEG data. The EEG signal quality was evaluated by examining the correlation between the prerecorded EEG and the EEG signals that were obtained by the dry EEG sensors. The linear correlation function toolbox from MATLAB (R2007a, The MathWorks, USA) was performed to calculate the signal differences that were measured by wet and dry sensors. Figure 8 shows the prerecorded EEG signals and the EEG signals that were measured by the proposed dry EEG sensors. The average correlation between the prerecorded EEG and the EEG signals recorded by the dry EEG sensor was very high, approximately 98.14%.

**Figure 9.**
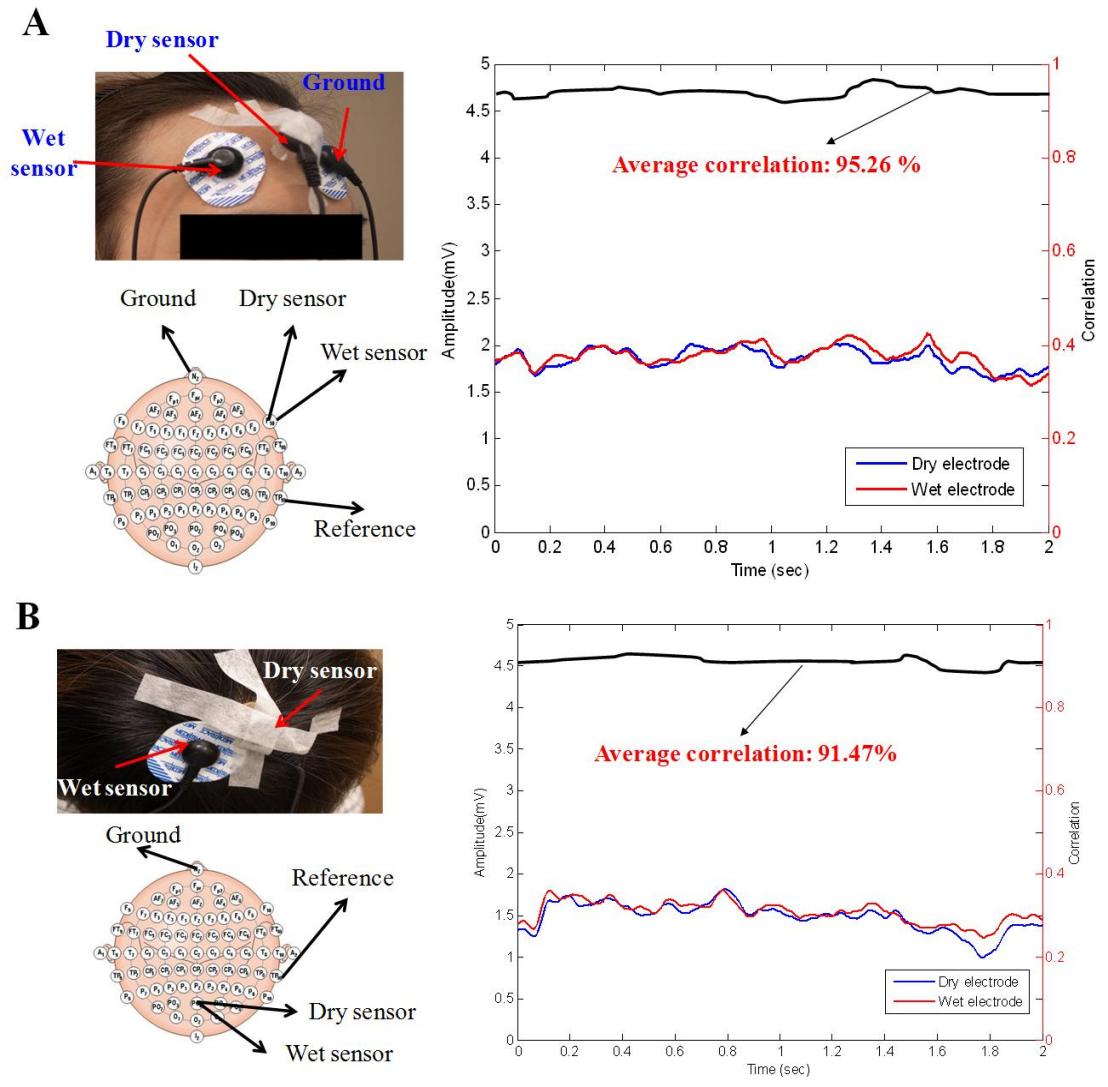
EEG signal comparison, as recorded by wet electrodes and the proposed dry sensors. (**A**) The EEG measurements on the forehead site (F10) and (**B**) the hairy site (POz) are presented.

**Figure 10.**
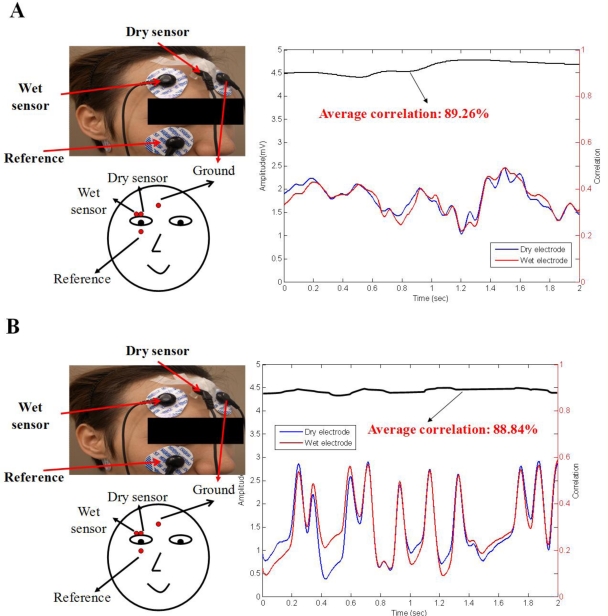
Signal comparison, as recorded by the wet electrodes and the proposed dry sensors. (**A**) The EOG measurements on a forehead site and (**B**) eye-blink signals on a forehead site are shown.

The signal quality was also compared between the conventional wet electrodes and the dry EEG sensor using the designed EEG readout circuit. Figure 9(A,B) show the results of EEG measurements using dry/wet electrode pairs on the forehead location (F10) and a hairy site (POz), respectively. Note that the wet electrode was used after scrubbing the skin. The recorded EEG signal correlations between the dry EEG sensor and the conventional wet electrode typically in exceeded 95.26% and 91.47% on forehead and hairy sites, respectively. Figure 10(A) shows the EOG and eye-blink signal measurements. For the EOG measurements, the correlation between the wet electrode and dry sensor was significant (over 89.26%). In Figure 10(B), the eye-blink signal correlation between the wet and dry sensors was approximately 88.84%. These results indicate that the signal quality recorded by the proposed dry sensor potentially achieves the same level of performance as the wet electrodes, without skin preparation or conductive gel usage.

## 4. Conclusions

In this study a novel dry EEG sensor was designed, fabricated, and experimentally validated for measuring EEG signals without any skin preparation. The advantages of dry EEG sensors can be summarized as follows: (1) they can achieve the necessary skin-sensor interface impedance to operate without any skin preparation or the use of conductive gels and furthermore, their impedance is even lower than that of wet electrodes on hairy sites; (2) spring contact probes and flexible substrates can buffer the electrode against an applied force; (3) they can be mass-produced using the injection molding process (the cost per unit will decrease with an increasing quantity of sensors manufactured); and (4) they can measure EEG signals on hairy sites.

The long-term impedance measurements suggest that this dry EEG sensor has the potential to provide very stable EEG signals. When compared with the other non-gel electrode types, the proposed dry sensors with spring contact probes and a flexible substrate can mold well to the scalp surface and increase the contact area of the skin-sensor interface to maintain low impedance. The dry EEG sensors showed promising and consistent EEG signal quality for all test subjects, unlike the wet electrodes. The EEG signal quality of these dry sensors showed long-term stability as well. Indeed, the proposed dry EEG sensor is ideally suited for future monitoring of human EEG states, especially for brain-computer interface applications, because no skin preparation or conductive gels are necessary, in contrast with the conventional wet contact-based electrodes.
